# Porosity prediction from well logging data via a hybrid MABC-LSSVM model

**DOI:** 10.1371/journal.pone.0335244

**Published:** 2025-10-27

**Authors:** Wei Su, Jie Gao, Wensheng Wu, Haoyu Zhang

**Affiliations:** 1 State Key Laboratory of Petroleum Resources and Prospecting, China University of Petroleum, Beijing, China; 2 Frontiers Science Center for Rare Isotopes, Lanzhou University, Lanzhou, Gansu, China; 3 School of Nuclear Science and Technology, Lanzhou University, Lanzhou, Gansu, China; Wadia Institute of Himalayan Geology, INDIA

## Abstract

Porosity is a key parameter for evaluating reservoir performance, but high-precision prediction is highly challenging in complex shale reservoirs due to the strong heterogeneity of the formation and the highly nonlinear relationship between logging parameters and porosity. Traditional prediction methods based on experience or physical models often have low generalization ability and accuracy. This study proposes a hybrid model (MABC-LSSVM) that combines a modified artificial bee colony (MABC) optimization algorithm with a least squares support vector machine (LSSVM) model. Inertia weights and acceleration coefficients are utilized to change the hyperparameters of the optimization model to achieve high-precision prediction of shale reservoir porosity using data-driven methods. The model inputs include compensating neutron log (CNL), density log (DEN), photoelectric absorption cross-section index (PE), and gamma ray log (GR) parameters. The proposed model is compared with the LSSVM, gradient boosting decision tree (GBDT), and ABC-LSSVM. The results show that the MABC-LSSVM model exhibits the best predictive performance. Its prediction results are highly consistent with the true porosity curve. The coefficient of determination (*R*^2^) is 0.93, significantly higher than for all comparison models. The findings demonstrate the effectiveness of combining an intelligent optimization algorithm with the LSSVM model. This approach is reliable for predicting the porosity in complex formations and performing reservoir evaluations in oil and gas exploration and development.

## 1. Introduction

The occurrence of shale gas differs substantially from that of natural gas. Its exploration and development are hot topics in the oil and gas industry. Porosity is a critical parameter for evaluating shale reservoirs and for quantitative research. It can be determined using laboratory analysis of core data. However, data loss occurs due to wellbore conditions, tool and instrument failures, improper storage, and incomplete logging data [1].

Mathematical or empirical equations can be established to determine reservoir physical parameters indirectly by utilizing the geophysical properties of rock formations based on logging data. A typical representative is the Archie equation [2], which is used to calculate reservoir physical parameters based on the relationship between rock RT and porosity, laying the foundation for interpreting logging data. Waxman and Smits [3] proposed the Waxman-Smits model to correct the influence of mud formations on resistivity. In addition to physical models, statistical analysis and empirical equations are common methods. In multiple linear regression analysis (MLA), a linear relationship is established between logging curves and core porosity [4]. Since empirical equations and statistical models are typically established for specific geological conditions, these models have low generalization ability [5] and do not consider the complex nonlinear relationship between reservoir parameters and logging data [6], which results in limited prediction accuracy.

Although many methods employ logging data to predict and evaluate reservoir physical parameters, the ambiguity of inversion problems, the solution stability, and the evaluation methods require further research. Model-driven inversion methods to determine the physical parameters of reservoirs depend highly on the physical model and the inversion algorithm. Since the applicability of these models is limited, the efficiency of global optimization algorithms is low. Therefore, we recommend using machine learning techniques to predict porosity from well logging data. Several studies have shown that machine learning methods outperform traditional methods in processing well logging data [7–9].

Machine learning methods are algorithms that enable artificial intelligence systems to learn from a small dataset and provide predictions. These methods have provided excellent results in well logging data processing and interpretation. Unlike traditional geophysical techniques, this method does not require establishing an explicit objective function. Instead, nonlinear implicit expressions between data and labels are created using iterative updates. Machine learning algorithms require a large amount of data, especially for predicting reservoir rock physical parameters. They are commonly used in geophysical applications [10,11]. Intelligent methods have been widely applied in the oil and gas industry for modeling and predicting reservoir parameters. Helle et al. [12] examined the combination of a backpropagation (BP) neural network and well logging data to predict porosity. One problem was that the model fell into the local optimum, which generally requires optimization algorithms. Mukherjee and Sain [13] applied AI techniques to predict reservoir parameters in gas hydrate sediments, demonstrating the capability of ML in modeling complex subsurface relationships. Malki et al. [14] combined a fuzzy logic algorithm with a BP neural network algorithm, and the prediction results were better than those of the BP neural network. Mukherjee et al. [15] proposed a petrographic classification framework using multiple ML algorithms trained on geophysical logs, which achieves high accuracy in lithological identification. Ahmadi et al. [16] employed genetic algorithms to optimize the parameters of an artificial neural network model to prevent the model from falling into a local optimum. Yasin et al. [17] combined a support vector machine (SVM) and particle swarm optimization (PSO) algorithm to predict the porosity of the Lower Goru reservoir in the Sawan gas field in Pakistan. Yang et al. [18] utilized a deep neural network (DNN) to predict sandstone porosity; the predicted and actual values were highly consistent. Nastaran et al. [19] adopted a convolutional neural network (CNN) model and achieved good accuracy in predicting porosity from seismic attributes, demonstrating the advantages of the CNN in dealing with nonlinear problems and reducing overfitting. Li et al. [20] combined a PSO algorithm with a long short-term memory (LSTM) network for lithology identification using well logging data. Their optimization approach was applicable to porosity prediction. Tong et al. [21] applied the least squares support vector machine (LSSVM) network to predict gas well productivity. These machine learning algorithms generally have higher prediction accuracy than rock physics models and geological parameter modeling [22]. These methods exist some limitations. First, the generalization ability of these models for complex geological formations is low. Due to different climatic and geographical conditions, the underground environment is complex and variable, especially in highly heterogeneous formations. Significant differences may exist in the logging data in the same formation. A general prediction model may have low prediction performance. Second, although machine learning methods better handle nonlinear problems than traditional methods, they are not without limitations. For example, the LSSVM requires suitable hyperparameters to achieve high performance. Finding the optimal hyperparameters is often complex and time-consuming, which creates a bottleneck for practical applications.

This paper proposes a combination of the modified artificial bee colony (MABC) optimization algorithm and the LSSVM to establish the MABC-LSSVM hybrid model. The innovative use of inertia weight and acceleration coefficient alters the search process, improving the model’s generalization ability. The penalty coefficient *γ* and the kernel parameter *σ* of the LSSVM model are optimized, preventing random parameter selection and improving the model’s prediction accuracy. The feasibility of using the compensating neutron log (CNL), density log (DEN), photoelectric absorption cross-section index (PE), and gamma ray log (GR) parameters to predict shale reservoir porosity is evaluated. Feature importance analysis is also conducted to increase the model’s interpretability.

## 2. Methodology

### 2.1. Least squares support vector machine (LSSVM)

**Fig 1:**
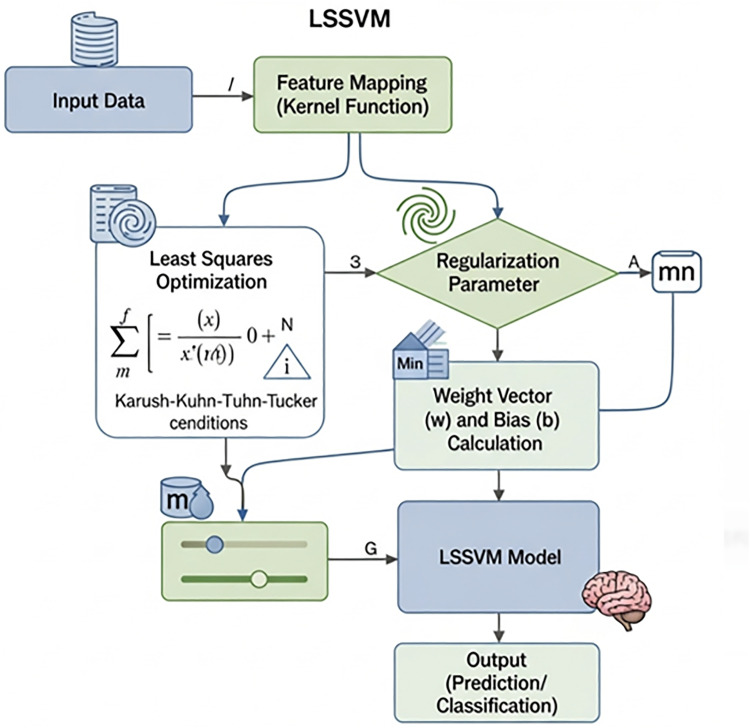
Schematic diagram of the LSSVM.

The LSSVM is a mature machine learning prediction method and an improvement of the SVM. Its largest advantage over the SVM is the fast training speed, good processing performance for a small sample size, and substantially lower computational complexity. The schematic diagram of the LSSVM model is shown in Fig 1.

We define the given sequence as a set of samples (*x*_*i*_,*y*_*i*_),*i* = 1,2,3,…,*N*, where *x*_*i*_ ∈ *R* denotes the input samples, and *y*_*i*_ ∈ *R* refers to the output data. Function φ(x) was used to map the samples to a high-dimensional feature space. The linear regression function is defined in Equation (1).


$$f(x)=w^{T}\varphi (x)+b$$ (1)

where w denotes the weight coefficient vector in the feature space. φ(x) denotes the kernel function for LSSVM. *b* ∈ *R* denotes the bias.

The evaluation is described as an optimization problem to ensure minimal structural risk:


$$\min g(w,e)=\frac{\text{1}}{\text{2}}w^{T}w+\frac{\gamma }{\text{2}}\sum_{i=1}^{n}{e_{i}}^{2}$$ (2)

$$s.t.y_{i}=w^{T}\varphi (x_{i})+b+e_{i}$$ (3)

where *e*_*i*_ is the error between the actual and predicted values. The penalty coefficient *γ* is greater than 0.

A Lagrangian transformation is used to solve the optimization problem:


$$L(w,b,e_{i},\alpha_{i})=g(w,e)-\sum_{i=1}^{N}\alpha_{i}\left[w^{T}\varphi (x_{i}+b+e_{i}-y_{i})\right]$$ (4)

The Karush-Kuhn-Tucker (KKT) conditions apply to the partial differentials (w, b, $e_{i}$, and $\alpha_{i}$), and $\alpha_{i}$ is obtained by the least squares method. The LSSVM regression function is expressed as:


$$y(x)=\sum_{i=1}^{N}\alpha_{i}K(x_{i},x_{j})+b$$ (5)

where $K(x_{i},x_{j})$ is the kernel function of the LSSVM. It significantly impacts the performance and generalization ability of the LSSVM model. A radial basis function (RBF) was chosen due to its strong generalization ability and flexibility, which results in more accurate and reliable model predictions. The RBF expression is presented in Equation (6):


$$K(x_{i},x_{j})=\exp(-\frac{{\left\|x_{i}-x_{j}\right\|}^{2}}{2\sigma^{2}}),\sigma >0$$ (6)

The kernel parameter σ and the penalty coefficient γ affect the LSSVM model’s performance [23], especially for a large samples size. The LSSVM performs matrix operations and kernel function verification in each iteration of the quadratic programming process to find the optimal solution, which is computationally complex. As the number of iterations increases, the squared terms grow, which leads to reduced computational speed and larger errors in later iterations.

Thus, it is necessary to optimize the two parameters using novel algorithms to compensate for the shortcomings of the LSSVM, which are affected by kernel functions and high dimensionality.

### 2.2. Modified artificial bee colony (MABC) algorithm

**Fig 2:**
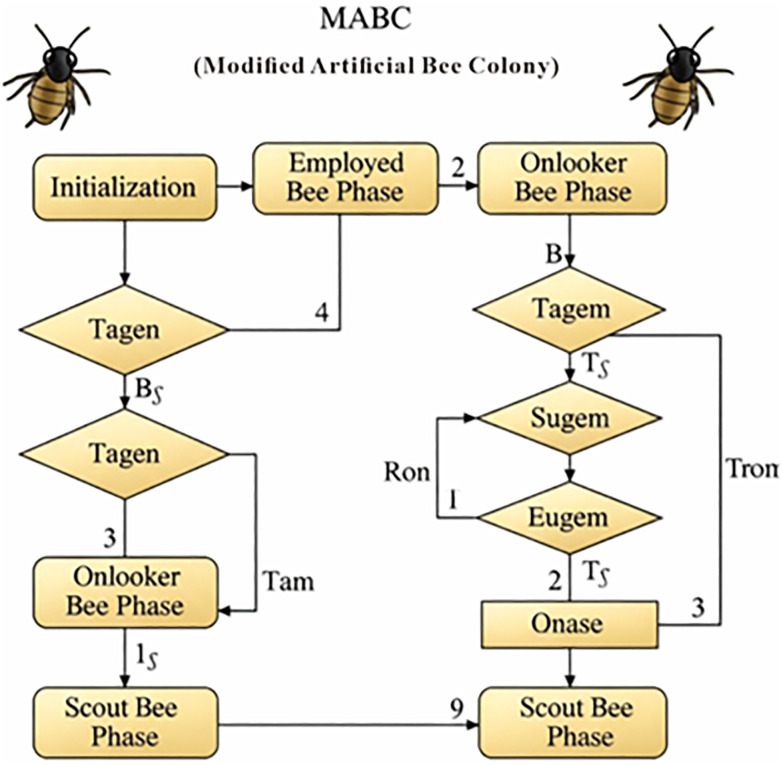
Flowchart of the artificial bee colony algorithm.


The ABC algorithm is a bionic intelligent computing method proposed by Turkish scholar for simulating bee colonies to search for nectar sources [24,25]. Its schematic diagram is displayed in Fig 2. The colony consists of employed bees, onlookers, and scouts. A bee’s position is represented by an M-dimensional vector *x*_*i*_ =[*x*_*i*1_, *x*_*i*2_, …, *x*_*i*M_], which represents a feasible solution *v*_*i*_=[*v*_*i*1_, *v*_*i*2_, …, *v*_*i*M_]. The number of employed and onlooker bees is equal, and both are denoted as *S*_*N*_.

$$v_{ij}=x_{ij}+\varphi_{ij}(x_{ij}-x_{kj})$$ (7)

where $i,k\in (1,2,\cdot \cdot \cdot ,S_{N})$ and j∈(1,2,···,M) are random numbers other than *j*. A random value controls the range of the neighborhood between j∈(1,2,···,M). This range decreases as the search approaches the optimal solution.

Onlooker bees evaluate information on the honey source shared by the employed bees in the dance area. They select the honey source based on the probability derived from Equation (7):


$$P_{i}=\frac{fit_{i}}{\sum_{j=1}^{S_{N}}fit_{j}}$$ (8)

where $fit_{i}$ is the fitness value of the *i*-th solution. The employed bee generates a new position using Equation (7) and makes greedy choices.

If a honey source location *x*_*i*_ cannot be updated in a pre-set number of cycles, it is assumed that this honey source does not exist. The scout bee randomly generates a new honey source location based on Equation (9) and replaces *x*_*i*_.


$$x_{ij}=x_{j\min}+rand(0,1)(x_{j\max}-x_{j\min})$$ (9)

where *x*_*ij*_ is the searched position corresponding to the *j-*th dimension of the *i*-th bee. *x*_*jmax*_ and *x*_*jmin*_ are the upper and lower bounds of the *j-*th dimension variable, respectively.

The search step size of the ABC algorithm is random, and the optimization speed is relatively slow. Therefore, the model falls into a local optimum or does not provide the global optimum. The search approach of the employed and scout bees is changed by using inertia weights and acceleration coefficients, enhancing the algorithm’s generalization ability. Equation (10) expresses the updating method:


$$v_{ij}=x_{ij}w_{ij}+2(\varphi_{ij}-0.5)(x_{ij}-x_{kj})\varphi_{1}+\varphi_{ij}(x_{j}-x_{kj})\varphi_{2}$$ (10)

where *w*_*ij*_ is the inertia weight. *x*_*j*_ is the best *j*-th parameter in this iteration. $\varphi_{ij}$ is a random number in the range of [0,1]. $\varphi_{1}$ and $\varphi_{2}$ are the acceleration factors that control the maximum step size. If the distance between the bees and the optimal solution is large, a large correction value is required to search for the global optimal solution. Small correction values are required for a small distance. We propose correction parameters for calculating new honey sources to enhance the bees’ search efficiency. The inertia weight and acceleration factor of the MABC algorithm during the search are defined as follows:


$$w_{ij}=\varphi_{1}=\frac{\text{1}}{\left(1+\exp(\frac{-fitness(i)}{ap})\right)}$$ (11)

$$\varphi_{2}=\left\{ \;\;\;\begin{array}{c} 1\\ \frac{\text{1}}{\left(1+\exp(\frac{-fitness(i)}{ap}\right)} \end{array}\right.$$ (12)

where *ap* is the fitness value in the first iteration. Different acceleration factors are utilized for the employed and scout bees to improve the algorithm’s convergence speed and generalization ability.

### 2.3. MABC-LSSVM model

The parameters and penalty coefficients of the LSSVM model are difficult to determine, affecting the accuracy of the model’s porosity prediction. The MABC algorithm was employed to optimize the parameters of the LSSVM model and enhance its prediction accuracy. The steps are as follows:

1) The logging data were preprocessed to extract features to predict porosity. The logging sequence was divided into training and testing sets.
2) Initial parameter values were chosen for the MABC-LSSVM model, including the nectar content, maximum number of cycles, and the number of cycles for termination.
3) The initial fitness value of the MABC algorithm was calculated and ranked. The root mean square error was the index to evaluate model fit.
4) Equation (8) was used to calculate the selection probability *P*_*i*_ of the honey sources. A roulette wheel selection was utilized for the onlooker bees to select the honey source, the probability of becoming the leader bee, and to search for new honey sources nearby.
5) Determine whether honey sources should be abandoned. If a honey source remained unchanged after the maximum number of cycles, it was abandoned, and the employed bee corresponding to the abandoned honey source became a scout bee. Equation (9) was utilized to generate a new honey source randomly to see if the stop iteration condition was met. If it was met, the optimal solution was output. Otherwise, Step 3 was repeated.
6) The optimal penalty coefficient and kernel parameter values were input into the LSSVM model. The MABC-LSSVM porosity prediction model was applied, and the predicted porosity was output.

**Fig 3:**
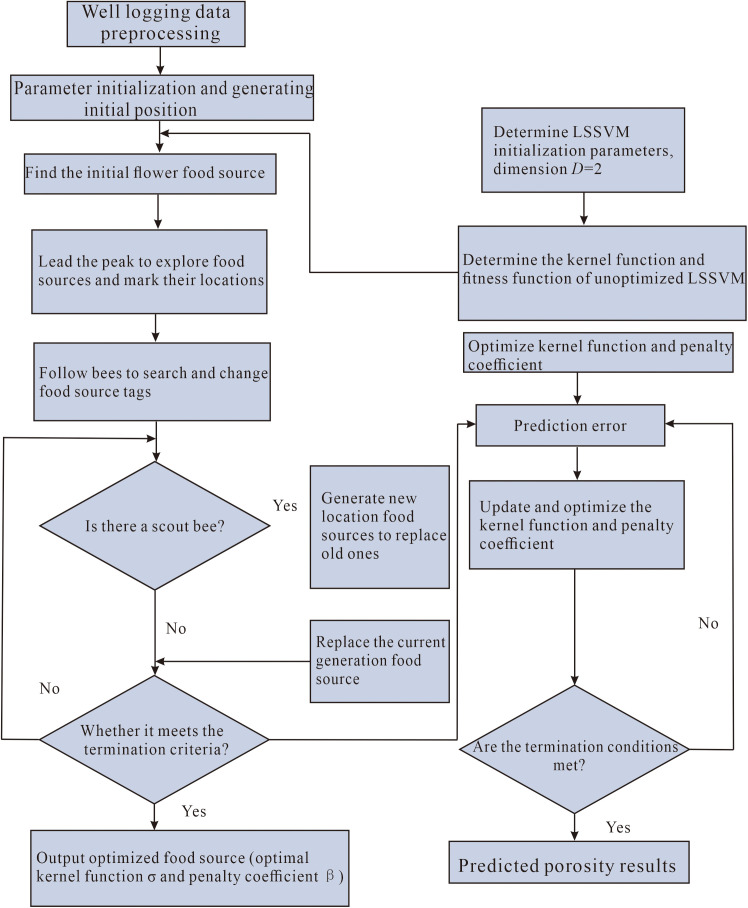
Flowchart of porosity prediction by the MABC-LSSVM model.

Fig 3 displays the steps of MABC-LSSVM model.

## 3. Data processing and model establishment

### 3.1. Data selection

The porosity and logging data were obtained from 12 wells at an uplift formation in the Qinshui Basin. This block was the sandstone reservoir with a porosity of 3.5% to 8.2% and a permeability of 1.527 × 10^−3^ to 745.23 µm^2^. The Qinshui Basin represents a typical medium-low permeability sandstone reservoir with characteristics common to many Chinese terrestrial basins. While specific to this geological context, the methodological framework developed here is transferable to other formations with appropriate calibration. Data processing, such as wellbore environment correction and inter-well standardization, was completed by the data provider. After basic data cleaning, 8,690 data points remained. Eight logging parameters were used as sample attributes: spontaneous potential (SP), gamma ray (GR), resistivity (RT), acoustic time difference (AC), compensating neutron log (CNL), density (DEN), photoelectric absorption cross-section index (PE), and borehole diameter (CAL).

**Fig 4:**
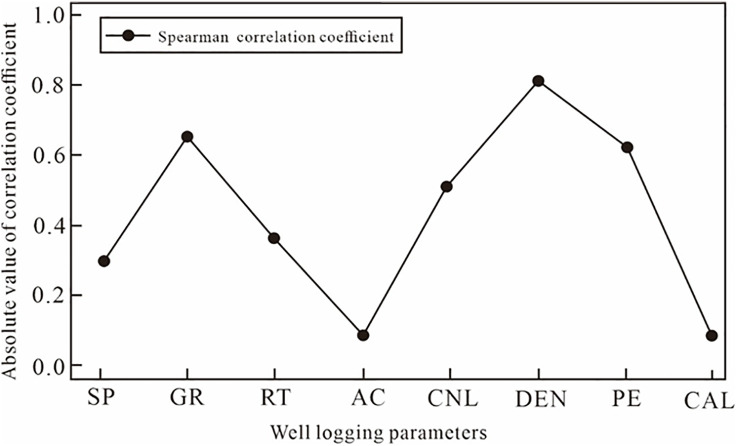
Spearman correlation coefficient.

**Fig 5:**
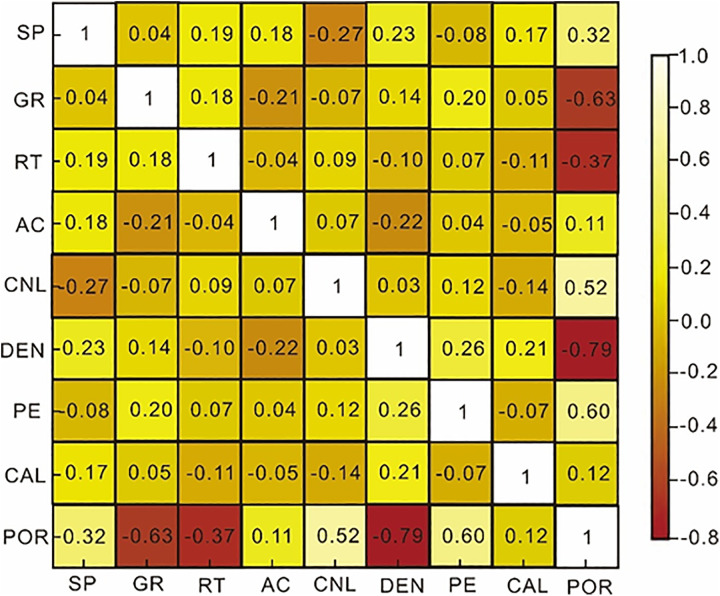
Heatmap of the Spearman correlation coefficient.


A complex nonlinear relationship exists between logging curves and porosity. To preliminarily assess feature-target dependencies, Spearman’s rank correlation coefficients were calculated (Figs 4-5). The results indicated that the correlation coefficients between the AC and the CAL are around 0.1, indicating a very low correlation with porosity. SP and RT exhibited relatively low correlation coefficients with porosity (|ρ|<0.4), whereas CNL, PE, GR and DEN showed higher absolute correlations (|ρ|>0.4). But correlation-based feature selection alone may oversimplify the problem by ignoring nonlinear interactions among features. Therefore, an *F*-test (analysis of variance) was conducted to further evaluate the statistical significance and contribution of each parameter to porosity prediction. The *F*-test quantifies how much variance in porosity can be explained by each logging feature compared with the residual variance. The Spearman’s correlation coefficient and the test statistic are computed as [26–28]:

$$\begin{array}{c} \rho =1-\frac{6\sum d^{2}}{n(n^{2}-1)} \end{array}$$ (13)

$$F=\frac{SSR/(k-1)}{SSE/(n-k)}$$ (14)

where ρ represents the Spearman correlation coefficient, with the values ranging from −1–1. A ρ value closer to 1 indicates a positive correlation between the two variables, and a value closer to −1 indicates a negative correlation. A value of 0 denotes no linear correlation between the two variables. *SSR* denotes the regression sum of squares, *SSE* denotes the error sum of squares, *k* denotes the number of test depth points, and *n* denotes the number of samples.

Higher *F*-values indicate stronger explanatory power for the corresponding feature. The test results (Table 1) revealed that CNL and DEN exhibited the highest *F*-values (>23), followed by GR and PE (8–15), while SP, RT, AC and CAL showed low significance levels (*F* < 5, *p* > 0.05), implying weak statistical association with porosity under the current data conditions.

**Table 1:** Results of *F*-test for input logging parameters.

	*F*-value	*p*-value
CNL	25.12	<0.001
DEN	23.42	<0.001
GR	12.15	0.002
PE	9.46	0.008
SP	4.03	0.057
RT	3.25	0.072
AC	1.76	0.172
CAL	1.29	0.166

Although SP and RT may still provide minor nonlinear contributions when used in ensemble models, their inclusion increased model complexity without improving predictive accuracy in this dataset. Consequently, CNL, PE, GR and DEN were selected as the optimal input features for subsequent model development. This combination of correlation analysis and *F*-test–based feature evaluation provides a more rigorous and interpretable approach for selecting sensitive logging parameters in porosity prediction.

### 3.2. Dealing with missing values and data division

Missing values and outliers frequently occur in logging data due to tool failures, borehole instability and other operational factors, which can significantly affect the performance of porosity prediction models. To address this issue, we analyzed the missing data patterns and found that most missing values followed a missing-at-random (MAR) mechanism. Such patterns, if not properly handled, can bias the distribution of features and degrade model generalization. Several common imputation strategies, including mean substitution, k-nearest neighbors (KNN)-based imputation, and multiple imputation were considered. Following the review of Xiong et al. [29], which summarized common imputation strategies, we adopted KNN-based imputation due to its effectiveness in preserving local feature structures in nonlinear geological data. Preliminary tests confirmed that it preserved feature fidelity and yielded higher predictive accuracy compared with simpler alternatives.

**Fig 6:**
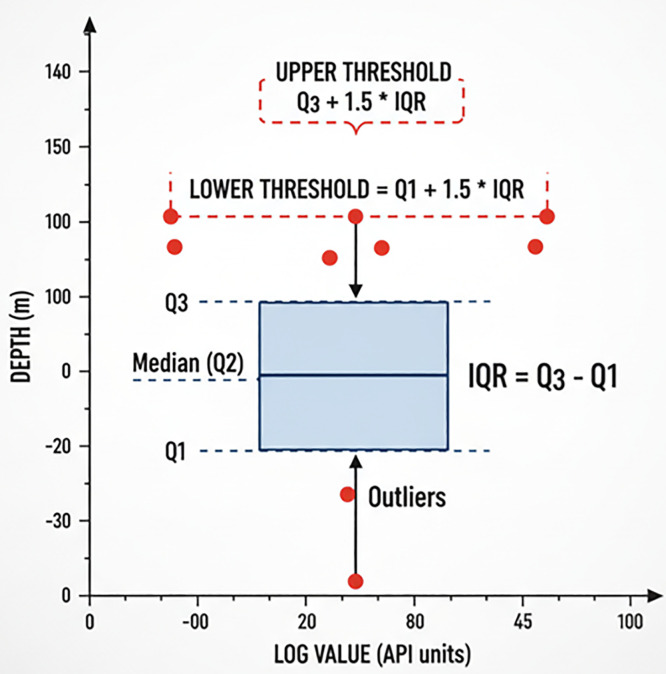
Box plot analysis schematic diagram.

For outliers, we applied a rigorous two-step procedure: interquartile range thresholds and boxplot analysis (Fig 6) were first used to detect anomalous values, and then geological constraints (such as the physical plausibility of porosity and density ranges) together with expert review were applied to distinguish spurious anomalies caused by measurement errors from genuine extreme values reflecting reservoir heterogeneity. The spurious anomalies were removed, whereas true extremes were retained. After these preprocessing steps, the processed logging data were normalized, randomly shuffled by row, and divided into training and testing sets with a 8:2 ratio. A portion of the processed data and corresponding porosity values is shown in Table 2.

**Table 2:** A portion of the dataset.

CNL (%)	PE (b/e)	GR (API)	DEN (g/cm^2^)	POR (%)
18.96	2.79	43.12	2.32	14.30
20.14	2.85	45.27	2.31	14.41
22.57	2.91	46.45	2.32	13.75
23.10	2.90	51.51	2.26	14.22
19.57	2.77	42.21	2.25	14.47

Various types of logging data have different dimensions and significant differences. Inputting these data without preprocessing can adversely affect the prediction results of porosity. The data were normalized using the maximum and minimum normalization functions. The input values had a range of [0,1]. The normalization is defined as follows:


$$X=\frac{x-x_{\min}}{x_{\max}-x_{\min}}$$ (15)

where *X* denotes the normalized data. *x* refers to the logging data. *x*_max_ and *x*_min_ are the maximum and minimum values, respectively.

A K-fold cross-validation technique was employed to improve the generalization performance of machine learning models [30]. The training dataset was divided into K equally sized folds (K = 5 for present analysis). One fold was randomly selected as the test set, while the remaining folds were used for model training. This approach allows all data samples to participate in both training and testing processes, ensuring that the performance evaluation is statistically reliable. In this study, a five-fold cross-validation strategy was adopted to assess the predictive accuracy of ML models, enabling a fair comparison under the same test protocol. According to the cross-validation procedure, no data were initially reserved exclusively for testing under the default setting. The entire dataset was divided into five equal folds, where one subset was used for model testing and the remaining four subsets were used for model training. Consequently, approximately 80% of the logging data were used for training, while the remaining 20% were used for model testing in each iteration. To ensure that each subset served once as the test set, the process was repeated five times. The average prediction error across all five folds was then calculated to represent the overall model performance.

### 3.3. Evaluation indicators

The LSSVM, gradient boosting decision tree (GBDT), and ABC-LSSVM models were compared. The standardized training data were input into the network model, and a grid was used to ensure the optimization of the hyperparameters. The test data were then used with the trained network models to compare their performances with that of the LSSVM-MABC model. The evaluation indices included the mean absolute error *(MAE*), *MSE*, and coefficient of determination (*R*^2^) to measure the prediction performance of the models. The *MAE* and *MSE* measure the degree of deviation between the predicted and true porosity. The smaller the value, the higher the prediction accuracy and the better the model performance. The *R*^2^ value describes the degree of agreement between the predicted value and the true value. The closer its value is to 1, the higher the goodness of fit and the better the predictive performance of the model [31,32]. The indices are calculated as follows:


$$MAE=\frac{\text{1}}{m}\sum_{i=1}^{m}\left|f_{i}-{\hat{f}}_{i}\right|$$ (16)

$$\begin{array}{c} MSE=\frac{\text{1}}{m}\sum_{i=1}^{m}{\left(f_{i}-{\hat{f}}_{i}\right)}^{2} \end{array}$$ (17)

$$\begin{array}{c} R^{2}=\frac{\sum {\mathrm{\backslash nolimits}}_{i=1}^{m}{\left({\hat{f}}_{i}-\bar{f}\;\right)}^{2}}{\sum {\mathrm{\backslash nolimits}}_{i=1}^{m}{\left(f_{i}-\bar{f}\;\right)}^{2}} \end{array}$$ (18)

where *f*_*i*_ represents the actual porosity of the *i*-th sample. $\hat{f}$ is the predicted porosity of the *i*-th sample. $\bar{f}$ is the average porosity, and *m* is the number of samples.

### 3.4. Model parameter settings

**Fig 7:**
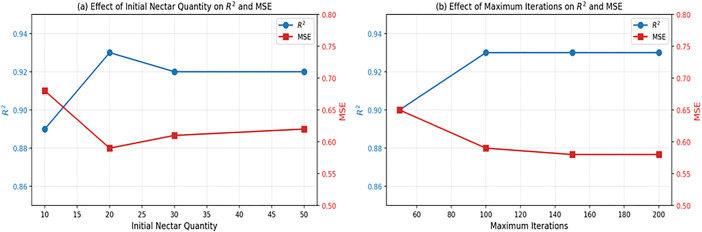
Effect of the initial nectar quantity and the maximum iteration number on model performance.

**Fig 8:**
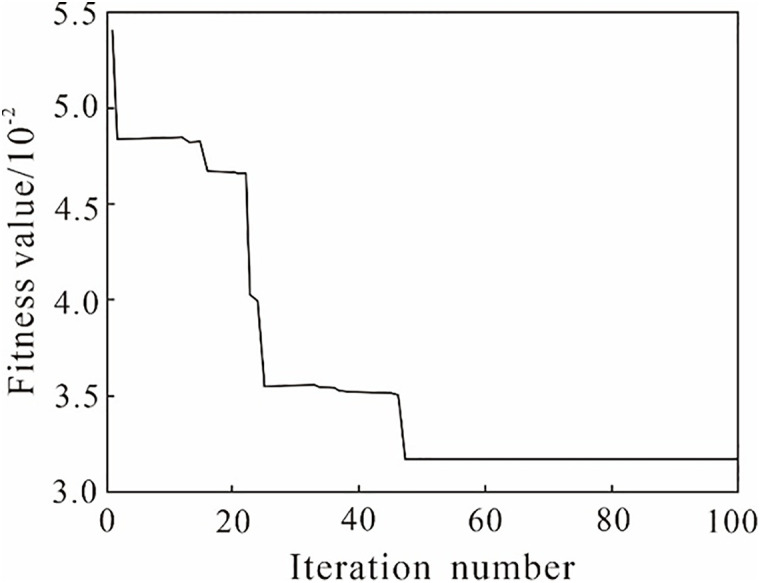
The fitness values during the MABC optimization.

The computer processor was an Intel (R) Xeon (R) CPU E5-2687W v4 @ 3.00GHz, the graphics card was an NVIDIA GeForce RTX 3090, the random access memory was 24 GB, the GPU acceleration library was CUDA11.1, and the deep learning framework was PyTorch. To show how sensitive the model performance is to initial hyperparameter choices, a sensitivity analysis of Fig 7 was conducted by the model’s prediction accuracy (**R*^*2*^*) and error (*MSE*). From Fig 7, it can be inferred when the nectar quantity increases from 10 to 20, the **R*^*2*^* value rises significantly (from 0.89 to 0.93), while further increases yield only marginal improvement. The model performance converges after about 100 iterations, with **R*^*2*^* remaining stable around 0.93. Therefore, an initial nectar quantity of 20 and a maximum iteration number of 100 provide the best balance between accuracy and computational efficiency. The other initial parameter settings of the LSSVM-MABC model are listed in Table 3. After the initial optimal parameters were set, *MSE* of the LSSVM model was calculated to determine the model fit of the MABC algorithm. Then, the fitness values during the MABC optimization are shown in Fig 8.

**Table 3:** Key initial hyperparameter settings for the model.

Hyperparameter	Setting
dimension	2
Parameter search range	[0.01, 1000]
Initial nectar quantity	20
Maximum iteration number	100
Number of iterations for termination	1000

The MABC algorithm obtained the optimal solution after 44 iterations, with a minimum MSE of 3.251 × 10^−2^ and a fast convergence speed. Subsequently, the fitness value stabilized. The MABC algorithm required only 6.897 seconds for 100 iterations. The optimal LSSVM neural network model parameters were a penalty coefficient of 48.521 and a kernel parameter of 8.547.

## 4. Results and discussion

### 4.1. Porosity prediction performance

**Fig 9:**
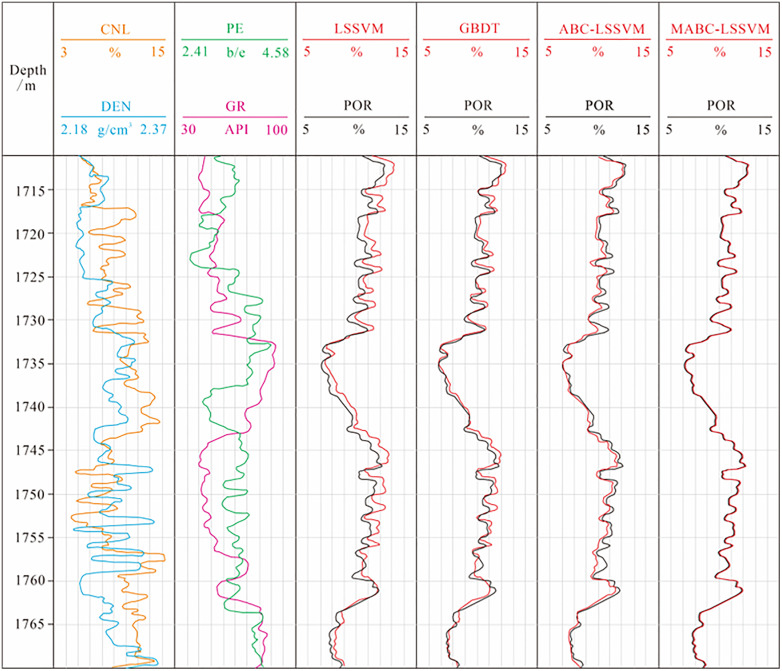
Porosity prediction results of different models.

The prediction results of the LSSVM, GBDT, ABC-LSSVM, and MABC-LSSVM models are presented in Fig 9. The black curve represents the true density value, which was obtained through geochemical methods from rock samples in the test dataset, with a sampling interval of 0.125 m. The red curves represent the prediction results of the four models.

A comparison of the four network models shows that all models achieve satisfactory performance in porosity prediction by using CNL, DEN, PE and GR parameters as sensitive feature parameters. Although some differences are observed between the models, the predicted curves generally match the measured porosity well, demonstrating the effectiveness and practicality of deep-learning–based porosity prediction from logging data. The curve obtained from the MABC-LSSVM model (red) almost completely overlaps with the true porosity curve (black), indicating minimal bias. This model accurately describes the sharp peak of the high-porosity reservoir interval at 1725 ~ 1735 m and precisely delineates the smooth baseline of the low-porosity, non-reservoir interval at 1755 ~ 1760 m, which exhibits excellent fitting performance and high sensitivity to formation heterogeneity. High-fidelity predictions are crucial for high-resolution reservoir characterization. Conversely, the porosity curve of the LSSVM model differs from the true curve, especially in regions with strong porosity fluctuations. The prediction does not describe abrupt changes and does not capture geological interfaces, systematically underestimating reservoir properties. The MABC-LSSVM outperforms the other three models, indicating a strong capacity for extracting meaningful information from logging data and superior generalization ability in porosity prediction.

The quantitative evaluation results (Table 4) reveal the performance levels of the four models. The three indicators (*R*^2^, *MAE*, and *MSE*) show highly consistent results. The MABC-LSSVM model exhibits the optimum predictive performance with the lowest error *(MAE* = 0.42, *MSE* = 0.59) and the highest goodness of fit (*R*^2^ = 0.93). The indicators suggest the model has very high prediction accuracy, the smallest variance, and high robustness. In contrast, the LSSVM model performs the worst, with the highest error (*MAE* = 0.89, *MSE* = 1.57) and the lowest *R*^2^ value (0.55), revealing an inability to capture the complex nonlinear relationship between the logging parameters and porosity under unoptimized conditions. The performances of the GBDT and ABC-LSSVM models are intermediate, and their error metrics and *R*^2^ values exhibit a significant stepwise improvement with increasing model complexity, confirming the effectiveness of model optimization.

**Table 4:** Comparison of porosity prediction accuracy for different models.

	*MAE*	*MSE*	*R* ^2^
LSSVM	0.89	1.57	0.55
GBDT	0.77	1.13	0.71
ABC-LSSVM	0.67	0.87	0.82
MABC-LSSVM	0.42	0.59	0.93

**Fig 10:**
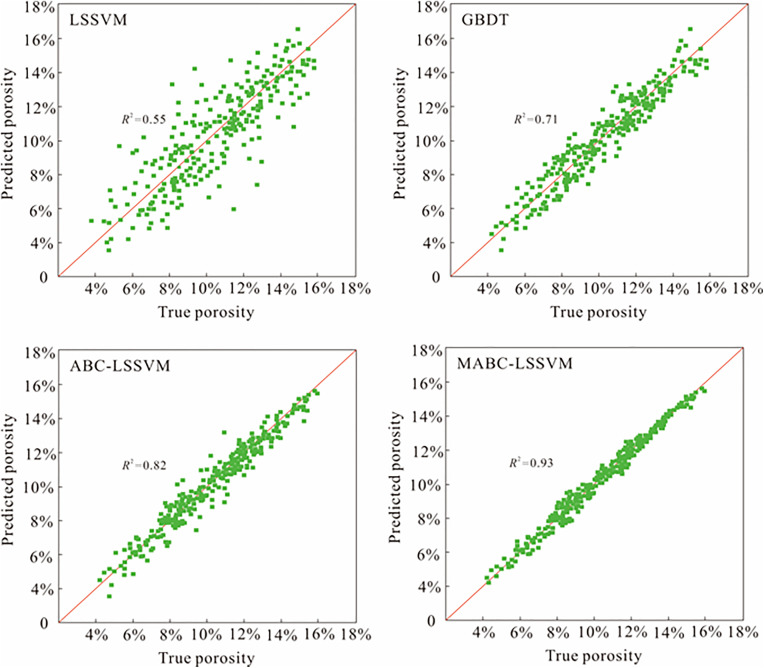
The prediction results of various models on the test set.

The scatter plot (Fig 10) depicts the quantitative results visually. The predicted porosity values of the MABC-LSSVM model are very close to the *y* = *x* line, which reveals a very small dispersion of data points. The *R*^2^ value is 0.93, demonstrating the high reliability and low uncertainty of the model’s prediction results. The scatter plots of the LSSVM, GBDT, ABC-LSSVM, and MABC-LSSVM models show significant randomness and dispersion. The data points are widely distributed on both sides of the diagonal, which indicates a high variance and a low confidence level. The low consistency of the prediction results means that these models provide incorrect assessments of the reservoir’s physical properties.

Multiple lines of evidence demonstrate the superior performance of the MABC-LSSVM model, which can be attributed to the optimization capability of the MABC algorithm. This algorithm effectively overcomes the inherent limitations of the conventional LSSVM model, including its sensitivity to hyperparameters and its tendency to become trapped in local optima. By employing an efficient global search strategy, the algorithm identifies the optimal hyperparameter combination, thereby enhancing the model’s generalization capacity and prediction accuracy. Developing a highly accurate porosity prediction model is crucial for both geological analysis and engineering practice. In contrast, models with low robustness, such as the traditional LSSVM, may lead to inaccurate reservoir performance evaluations, ultimately compromising reservoir modeling and drilling scheme design. The high-precision porosity predictions achieved by the MABC-LSSVM model provide a reliable data foundation for accurate geological modeling and reservoir evaluation, substantially reducing the risks and uncertainties associated with exploration and development.

### 4.2. Model practicality

**Fig 11:**
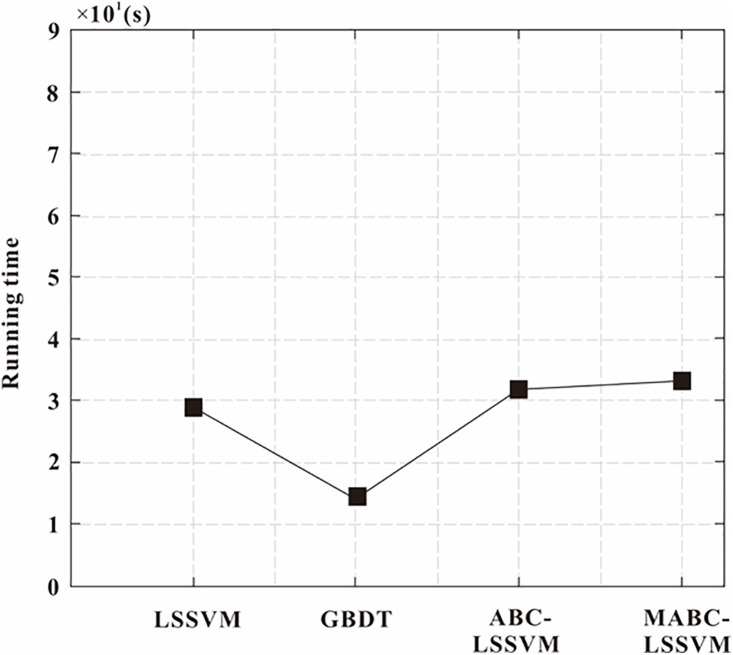
Comparison of the running times of different models.

Deep learning methods for predicting formation porosity have higher operational efficiency than conventional methods. The proposed porosity prediction method based on deep learning has low computational complexity, which is represented by FLOPs value. The running time of the model with the optimal hyperparameter settings was evaluated on the above dataset. The results are shown in Fig 11. Table 5 lists the size and FLOPs value of different models.

**Table 5:** The size and FLOPs value of different models.

Model	Model size/M	FLOPs (computational complexity)
LSSVM	17.25	48.72
GBDT	20.14	37.45
ABC-LSSVM	32.21	52.21
MABC-LSSVM	31.24	52.89

The MABC-LSSVM and ABC-LSSVM models involve the largest number of training parameters, as they require multiple iterations to determine the optimal hyperparameters. Each iteration requires the training of the LSSVM model. In contrast, the GBDT model exhibits the lowest computational complexity (FLOPs value) since it only involves tree traversal. However, the LSSVM and its optimized variants must compute kernel functions for all training samples, which can be slower than GBDT when the dataset is large. Consequently, GBDT demonstrates higher computational efficiency during both training and prediction. While the MABC-LSSVM achieves the highest prediction accuracy, it also incurs the greatest computational cost—a trade-off that is often acceptable in earth science and engineering applications where high-precision prediction is essential.

### 4.3. Feature importance

**Fig 12:**
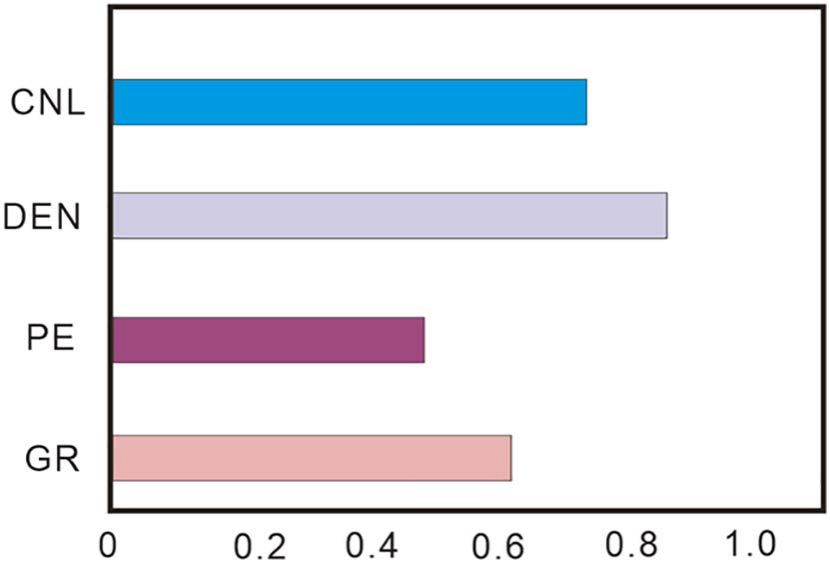
Schematic diagram of the feature importance.

The Shapley Additive exPlanation (SHAP) method was employed to conduct feature importance analysis of MABC-LSSVM model to quantify the contributions of logging parameters to porosity prediction [33]. The SHAP method is based on cooperative game theory and decomposes the model prediction results (i.e., porosity prediction values) into the sum of the contributions of the input features (CNL, DEN, PE, GR), which provides a highly transparent model interpretation in Fig 12.

The SHAP analysis was applied to the MABC-LSSVM model with the best performance. It provided the global importance ranking of the input features and the impact direction. The results indicate that the ranking of the average impact of the logging parameters on porosity prediction from high to low is DEN > CNL > GR > PE. This ranking result is highly consistent with classical rock physics theory, verifying the consistency between the information learned by the model and the principles of geological science.

### 4.4. Limitation

The contributions of this study lie in analyzing the feasibility of using CNL, DEN, PE, and GR logging parameters to predict porosity with the MABC-LSSVM model and in demonstrating the superior performance of the proposed model. This model showed excellent performance for learning the complex and nonlinear relationship between the logging parameters and porosity. It did not fall into the local optimum and exhibited high prediction accuracy in unknown well sections, demonstrating its high robustness.

Despite the model’s superior performance in predicting porosity, it has significant limitations that cannot be ignored in practical applications. The model performance is highly dependent on the quality and representativeness of the training data. If the training data do not represent all types of lithology, fluids, and pores of the target formation, the prediction accuracy may be significantly lower. Second, this model typically only provides a single predicted value and does not quantify prediction uncertainty. Accurately evaluating the confidence interval and uncertainty of porosity prediction is crucial for risk assessment and estimating reservoir properties in oil and gas exploration. The MABC-LSSVM model does not provide probabilities, which may be a disadvantage in engineering decisions that require consistent accuracy and risk. Future studies could incorporate data with high variability to develop a more powerful and applicable model for most reservoirs.

## 5. Conclusions

This study analyzed the feasibility of predicting the porosity of shale reservoirs using multiple machine learning models and logging parameters. A hybrid LSSVM model-MABC optimization algorithm (MABC-LSSVM) was proposed to enhance the prediction accuracy. The CNL, DEN, PE, and GR logging parameters were the model inputs, and the proposed model was compared with the LSSVM, GBDT, and ABC-LSSVM models. The following conclusions were drawn:

1) The inertia weight and acceleration coefficient were incorporated into the ABC algorithm to change the search process and improve the model’s generalization ability. The penalty coefficient *γ* and kernel parameter *σ* of the LSSVM model were optimized using the MABC, reducing the randomness of parameter selection of the LSSVM model and increasing the prediction accuracy.
2) The MABC-LSSVM model demonstrated outstanding predictive performance on the test set. The prediction results were highly consistent with the real porosity curve. The *R*^2^ value was 0.93, and the *MAE* and *MSE* values were 0.42 and 0.59, respectively. These results were superior to those of the comparison models, especially the LSSVM model, whose *R*^2^ value was only 0.55 with the highest prediction error.
3) This research demonstrates the effectiveness of combining intelligent optimization algorithms with machine learning models in handling complex geological-geophysical inversion problems. The proposed method is reliable for high-precision and high-resolution reservoir parameter prediction. The highly accurate porosity prediction results provided by the MABC-LSSVM model offer a solid data foundation for precise geological modeling and reservoir assessment, reducing risks and uncertainties in oil and gas exploration and development.

Although the MABC-LSSVM model achieved excellent results, it has some limitations. These limitations include high computational complexity in the training stage, strong dependence of prediction results on the quality of training data, and the inability to provide quantitative information on the uncertainty of the prediction results. Future studies should select training data with high variability to establish a more powerful model that is applicable to most reservoirs.

## Supporting information

S1 Data All Experimental dataset. (XLSX)
